# SoftSAR: The New Softer Side of Socially Assistive Robots—Soft Robotics with Social Human–Robot Interaction Skills

**DOI:** 10.3390/s23010432

**Published:** 2022-12-30

**Authors:** Yu-Chen Sun, Meysam Effati, Hani E. Naguib, Goldie Nejat

**Affiliations:** 1 Autonomous Systems and Biomechatronics Laboratory (ASBLab), Department of Mechanical & Industrial Engineering, University of Toronto, Toronto, ON M5S 3G8, Canada; 2 Toronto Smart Materials and Structures (TSMART), Department of Mechanical & Industrial Engineering, University of Toronto, Toronto, ON M5S 3G8, Canada; 3 Institute of Biomedical Engineering, University of Toronto, Toronto, ON M5S 3G8, Canada; 4 Toronto Institute of Advanced Manufacturing (TIAM), University of Toronto, Toronto, ON M5S 3G8, Canada; 5 Toronto Rehabilitation Institute, Toronto, ON M5G 2A2, Canada; 6 Rotman Research Institute, Baycrest Health Sciences, North York, ON M6A 2E1, Canada

**Keywords:** socially assistive robots, soft robotics, human–robot interaction

## Abstract

When we think of “soft” in terms of socially assistive robots (SARs), it is mainly in reference to the soft outer shells of these robots, ranging from robotic teddy bears to furry robot pets. However, soft robotics is a promising field that has not yet been leveraged by SAR design. Soft robotics is the incorporation of smart materials to achieve biomimetic motions, active deformations, and responsive sensing. By utilizing these distinctive characteristics, a new type of SAR can be developed that has the potential to be safer to interact with, more flexible, and uniquely uses novel interaction modes (colors/shapes) to engage in a heighted human–robot interaction. In this perspective article, we coin this new collaborative research area as SoftSAR. We provide extensive discussions on just how soft robotics can be utilized to positively impact SARs, from their actuation mechanisms to the sensory designs, and how valuable they will be in informing future SAR design and applications. With extensive discussions on the fundamental mechanisms of soft robotic technologies, we outline a number of key SAR research areas that can benefit from using unique soft robotic mechanisms, which will result in the creation of the new field of SoftSAR.

## 1. Introduction

Robotics has been constantly evolving to encompass new research methods and applications. Robots have entered our lives through everyday applications in warehouses [1], surveillance and search and rescue [2], self-driving cars [3], retail and grocery stores [4], and healthcare [5]. Today, robots and humans are engaging in more complex tasks that require direct interaction between them in shared environments, resulting in the emergence and growth of the field of human–robot interaction (HRI) [6]. 

HRI is a unique multidisciplinary field, which incorporates robotics, artificial intelligence, human factors, and the social sciences [7]. Robots can engage in either or both physical and/or social HRI. Robots deployed for physical HRI (pHRI) generally perform manipulation tasks in collaboration with humans or consist of wearable devices, such as exoskeletons, to help people achieve certain functions [8,9]. Social HRI (sHRI) encompasses interactions with people using social cues and social norms, including a combination of both verbal and nonverbal communication modes [10,11]. 

The term socially assistive robots (SARs) was first defined by Feil-Seifer et al. [12] as social robots that integrate assistive capabilities with interactive capabilities. SARs mainly engage in social HRI to provide aid to people with numerous tasks from social and behavioral skill learning to assistance with activities of daily living through prompting, encouragement, and reinforcement [13,14,15]. SARs provide support to our most vulnerable populations, which include a wide range of users from children with autism spectrum disorder or cognitive disorders [16,17] to older adults living with cognitive impairments or recuperating from a stroke [13,14]. They also address depression, loneliness, and social isolation through companionship [18,19,20].

One of the major limitations in soft-bodied SAR is the safety concern during pHRI. As a result, complaint designs can be incorporated into SARs in addition to the passive soft materials to address the limitations of existing soft-bodied SARs by reducing and absorbing the impact during pHRI, thus, protecting both the human and the robot [21,22]. In contrast, the new proposed SoftSAR technology will incorporate not only the passive impact absorption capability of the soft material body and compliant mechanisms but also utilize novel smart material-based actuation/sensing technologies to replace the traditional motor-based movement and rigid body frames/linkages. Such integration will result in improvements in SAR functionalities, such as improved emotion expression, through soft actuation instead of motor-controlled robotic-like motion.

What is unique about the HRI scenarios for SARs is that they can interact both socially and physically with people when needed using a variety of interaction modes. For example, for social HRI, their nonverbal communication modes can extend beyond the traditional human-like modes to encompass changes in color and shape to convey their intent [11]. With respect to physical contact, some SAR embodiments welcome touching, petting, or hugging [21]. In order to successfully carry out these interactions, it is important for SARs to have self-awareness, empathy, and be adaptable to the users and environments they interact with. 

We identified a new research collaboration for SARs that uniquely encompasses the utilization of soft robotic technologies to enhance the HRI experience. We term this new field **SoftSAR (soft robotics + SAR).** The main objective of this perspective manuscript is to explore how the two fields of SARs and soft robotics can benefit and complement each other, while proposing new cross-disciplinary research ideas. SAR research has typically focused on the appearance and behaviors of interactive robots in order to engage users in different assistive tasks, while detecting human affect and behaviors as feedback. In this manuscript, we discuss the potential benefits of using soft robotic actuator mechanisms for next-generation SARs to: (1) uniquely show robot intent and emotions through the use of reconfigurable shapes and colors; (2) provide tactile feedback via the use of soft and texture-changing electronic skin; and (3) inspire biomimicry of human-like gestures using inflatable elastic material with specifically designed pneumatic networks. In addition, new functionalities, such as active sensing and self-healing ability, of soft robots are also discussed. For the SAR research community, this manuscript provides a detailed discussion on the characteristics, properties, and the advantages of incorporating soft robotics within SAR design, from the perspective of novel sensing and actuation abilities. On the other hand, this manuscript is also of interest to the Soft Robotics community as it highlights the inclusion of HRI considerations during the robotic design process in order to introduce new societal and human-friendly applications, which are often overlooked for material advances.

## 2. Soft-Bodied SARs

The majority of SARs are designed with life-like embodiments that range from character or animal like to human like. They can use soft materials for their outer shells or even individual body parts. HRI can be influenced by a robot’s appearance, the materials used to develop it, and its overall functionality [22]. These robots utilize soft shells for two primary reasons: (1) to mimic biological features, such as skin or fur, for adding realism [23,24] and (2) to improve acceptance by having an aesthetic and biomimetic appearance [25]. Soft and elastic materials, such as silicone [26,27] and foam [28], have been used to construct the outer shells of SARs to achieve realism, softness, and also to absorb impact from high-force physical interactions [21,29]. 

Louie et al. [30] used the human-like SAR Brian 2.1, with a deformable silicone face that could display various natural facial expressions (e.g., happy, sad), in combination with vocal intonation and gestures and body language to assist older adults, including those with mild cognitive impairments, in their everyday activities, such as meal eating and memory games. Wood et al. [27] developed a child-sized humanoid assistive robot, Kaspar, for robot-assisted therapy with children with autism. The robot’s head and two arms are made from soft silicone, RoboSkin, with embedded tactile sensors used to detect different kinds of touch, such as finger poking or hand grasping [31]. Saldien et al. [28,32] developed a robot, Probo, for interactions with hospitalized children. Probo has a movable trunk made from foam used to display different emotions.

Animal-like SARs have mainly encompassed furry bodies that are inviting to touch and/or being held. For example, Moyle et al. [33] used a seal-like robot, Paro, for animal therapy in nursing homes, where real animals were restricted due to infections and allergies. Paro resembles the appearance of a baby harp seal and has soft white artificial fur that covers a hard skeleton [34]. Tactile pressure sensors made from a dielectric material are used beneath the robot’s fur shell to respond to stroking or even harsh petting by moving its tail and opening/closing its eyes. Paro can also detect being held via posture sensors [35]. Stiehl et al. [36] developed the Huggable teddy bear robot for pediatric care. The robot has a soft silicone-based skin with fur. The robot has full-body sensitive skin containing potentiometers, thermistors, and force and electric field sensors to detect kinesthetic, temperature, touch, and pain information [18,37]. Kozima et al. [26] developed Keepon, a small chick-like robot for HRI with young children, including those with developmental disorders and autism. The robot has a hollow body made from silicone rubber, which allows it to be easily deformed during touch interactions. Such an exterior also safely shields children from all the robot’s internal electronics, including DC motors and circuit boards. During HRI, Keepon has the ability to communicate with users by orienting its head in a certain direction, simply rocking left and right or bobbing up and down through its deformable body.

## 3. The Use of “Soft Actuation” in SARs

**Figure 1:**
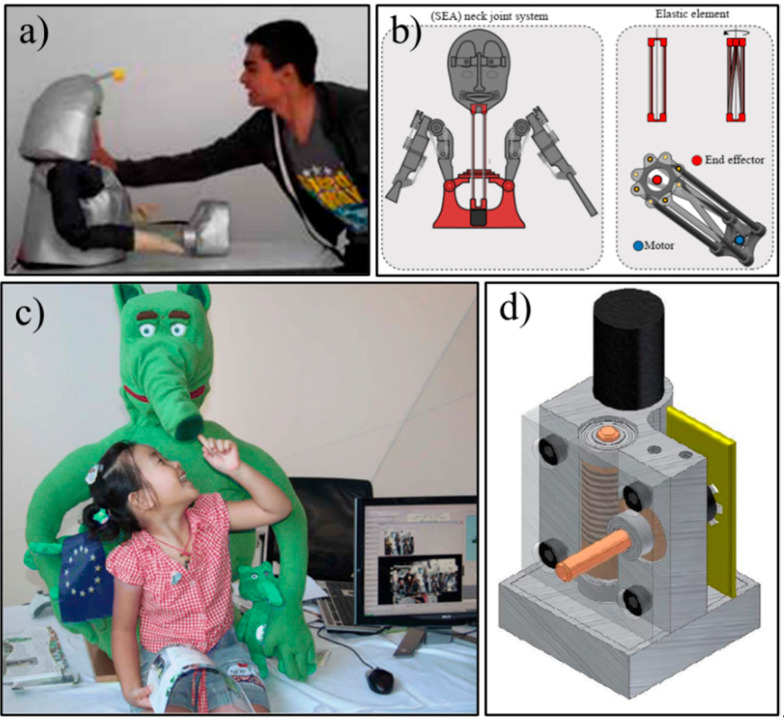
(**a**) The CASTOR robot’s soft outer shell has the ability to absorb impact, courtesy of Casas-Bocanegra et al. [21], (**b**) the SEA neck joint and the integrated element connecting the head and the body in the CASTOR robot, courtesy of Casas-Bocanegra et al. [21], (**c**) the Probo robot with a removable soft jacket and expressive trunk, courtesy of Saldien et al [38], and (**d**) the schematic of the NBDS actuation system for Probo showing the worm gear design, courtesy of Saldien et al [32]. All images are courtesy of open access articles.

To date, limited research has discussed the utilization of “soft actuation” for the design of SARs [21,29]. Namely, only a handful of SARs exist that have a combination of a soft body and a soft-actuation mechanism. Herein, soft actuation is achieved by using flexible and compliant mechanisms at the robot’s joints and linkages where motions are being generated. Casas-Bocanegra et al. [21] developed the character-like robot CASTOR (CompliAnt SofT Robotics), Figure 1a, with both a soft body made from flexible material thermoplastic polyurethane (TPU) and a soft-actuation system. The soft body and the soft-actuation system were designed to absorb impact and high force to improve the overall safety, in contrast to the SAR with hard shell and rigid linkages, during the pHRI, as both features have the ability to passively absorb impacts from users and large forces applied by the user during pHRI, ensuring a safe pHRI interactive scenario. The actuation mechanism incorporates soft actuators with compliant mechanisms by using “series elastic actuators” (SEAs) in the robot’s neck joint, as shown in Figure 1b. In the SEA design, an elastic element is used to connect the robot’s head to the servomotor to create neck motions, such as head shake and left/right turning. The elastic element consists of four aluminum bars attached to two 3D-printed polylactic acid (PLA) parts, fabricated via the fused deposition modelling (FDM) technique, to allow for compliant motion in the rotational and axial directions. Such a design allows the robot to change its elasticity at the neck joint (Figure 1b) based on the amount of external force applied during pHRI. Due to such a feature, the users will not experience the reaction force from the joint, thus, improving the overall safety during pHRI. 

Saldien et al. [28,29] introduced a robot, Probo, which consists of an outer layer made from a removable plush fur jacket covering a polyurethane foam layer, as shown in Figure 1c. To achieve safe and soft movements, two compliant actuators (the compliant Bowden cable-driven actuator (CBCDA) and the non-back drivable servo (NBDS)) were utilized in the robot [29]. The CBCDA is a passive compliant servomotor system that transmits motions over long distances by using cables (the Bowden cable). The main actuators (servomotors) in the CBCDAs are placed in the body of the robot while the cables are connected to several motional expression components, such as the eyes, eyelids, and the eyebrows, in the facial region. As the cable itself is highly flexible and elastic, compliance of the CBCDA can be achieved by adjusting the tension of the Bowden cable during the winding process to the servomotor. Both the flexible nature of the cable and adjustable tension feature enable the CBCDA to act as damping features to absorb a certain amount of force during physical interaction, such as punching or pushing from the users. On the other hand, the NBDS uses a set of worm gear and worm wheel design, as shown in Figure 1d, to ensure the transmission from the servo is non-reversible. This non-reversibility ensures that the output (physical force applied by children interacting with the robot) will not be transmitted back to the motor or the robot. On the other hand, the NBDS uses a set of worm gear and worm wheel design, as shown in Figure 1d, to ensure the transmission from the servo is non-reversible. This non-reversibility ensures that the output (physical force applied by children interacting with the robot) will not be transmitted back to the motor or the robot. One of the noticeable features of Probo is its movable trunk, made from foam, while the trunk motions are generated by the NBDSs, ensuring safe pHRI. In addition, NBDSs are also implemented for controlling the robot’s ears and mouth, which are used for the emotion expressions.

## 4. Soft Robots and Current SAR Designs

The term soft robot has been mainly used to describe SARs with either soft shells or bodies and/or with compliant or soft actuation. However, the overall structure and the majority, if not all, of the linkages and the core structures used in the design of existing SARs are still rigid. In the present day, the term “soft robotics” represents an emerging and important research field outside of the aforementioned definition used in SAR research. Namely, “soft robotics” refers to the design, control, and fabrication of robots comprising soft/compliant materials, with a special focus on compliance and deformation of the materials during the interaction with the surrounding environment, instead of rigid components utilized in traditional robots [39]. In addition, soft robotic research also includes the development of bioinspired continuum robots, which exhibit deformability across their bodies [40]. Soft robots are mainly composed of flexible materials, such as elastomers, that are similar to soft biological materials [41]. The use of these materials enables them to undergo continuum motions [42]. Furthermore, actuation is achieved via nonconventional mechanisms rather than electric components, such as electric motors or linear actuators. The motions of soft robots are accomplished by using responsive smart materials for which movements are realized by their intrinsic material properties [42]. Compared to traditional actuation systems, such as servomotors, the modern bioinspired soft robots offer advantages, such as high flexibility and deformability in their designs [42,43]. Another unique characteristic of soft robots is their high degrees of freedom (DOFs). Traditional robots, including SARs, have a certain number of DOFs based on the number of motors, actuators, and rigid linkages used. However, soft robots can have DOFs close to infinity due to redundancy in DOFs in their continuum design [44], which allows soft robots to bend through a series of continuous arcs instead of pre-defined joints [45]. Many soft robots also use soft sensing to perceive their environment. In order to adhere to the dynamic motion of their soft body, these sensory elements are also made from flexible materials [46]. Based on their stretchable nature, the sensors enable the soft robots to detect their own positions and contact forces during interactions with objects [47]. A summary comparison between traditional robots and soft robots can be found in Table 1.

Based on the aforementioned modern definition of the soft robotics field, the “soft exterior/body” and “soft actuation” considered for the limited number of SARs, such as the works presented in [21,28,29], do not directly fit within the scope of “soft robotics. This is due to many factors. Firstly, the designs of SARs often do not use continuum actuation throughout the robot’s body. Secondly, their DOFs are limited due to the presence of rigid linkages and use of electric motors. Thirdly, soft-structure-related works [21,28,29] were made from compliant materials (TPU), and compliant mechanisms (CBCDA/NBDS) were mainly utilized for passive damping to minimize the impact to the robot and user during harsh physical contact, while actuation movements were achieved by servomotor control. Lastly, the traditional sensory components of SARs often focus on unimodal sensory inputs (i.e., the touch signals from users), while soft robotic sensors or artificial skins offer advantages, such as the ability for multi-modal sensing (pressure, strain, temperature, etc.) with the addition of superior flexibility over mechanical-based sensors/transducers [48]. Therefore, we explore the potential inclusion and benefits of soft-robotic technologies to SARs by first investigating the state-of-the-art in soft robotics.

## 5. Soft Robotics: Towards “Soft” Actuation and Sensing

Soft robots are adaptable and robust to various unstructured and unpredictable environments: they are able to operate at undefined narrowed paths with limited space or deep-sea environments with water pressure; they are also able to maneuver in different adverse terrestrial conditions, including, but not limited to, snow, uneven surfaces, and water puddles [49,50,51]. To be able to function in dynamic environments, the inclusion of soft actuators and sensors is key in the design of these adaptable robots. Soft actuators are soft material-based systems that have the ability to undergo large changes in their geometries in response to different stimuli, such as pneumatic, mechanical, thermal, and electrical [52]. On the other hand, soft and compliant sensors are made from soft and flexible materials, which provide sensory signals to the soft robots based on the amount of stress or deformation applied to them [42]. A detailed discussion of available soft actuators and flexible sensors is provided herein in addition to highlighting the important principles used in these technologies to determine how they can be used by SoftSAR systems. 

### 5.1. Soft Actuation

Many SARs need to physically move their overall body or body parts in order to express their emotions and intent through the use of facial expressions or gestures and body language, whether it be through the individual or combined motion of their face and head, torso, arms, and/or legs [11]. This has been achieved mainly through the use of DC motors [26] and servomotors [29]. Alternatively, soft actuation can be achieved by three distinct mechanisms [42]: (1) pressurized fluidic and pneumatic-driven actuation, (2) variable-length tendon-driven actuation, and (3) electroactive polymeric (EAP) actuation, which can be further categorized into electronic or ionic subgroups, depending on their actuation mechanisms and stimuli. Electronic-based EAPs, such as dielectric elastomers, are able to produce movements within milliseconds and have high energy density, between 0.01 and 3 MJ/m^3^. However, a high electric field (70–400V/μm) is required [42]. On the other hand, ionic-based EAPs require the presence of an aqueous environment or a liquid-filled medium in order for the ionic movement to take place, which triggers the actuation [42]. Based on such constraints, both types of EAPs are not suitable for SARs in their current state: the high electric field requirement imply the need for a high-voltage power source, which may have safety concerns for SARs. Similarly, SARs do not operate in an aqueous or solution-rich environment for ionic diffusion to take place. These three types of actuations have their own unique characteristics in providing desired motions, which are discussed with respect to their advantages and limitations below. A summary of different soft-robotic technologies can be found in Table 2.

#### 5.1.1. Pressurized Fluidic and Pneumatic-Driven Actuation

**Figure 2:**
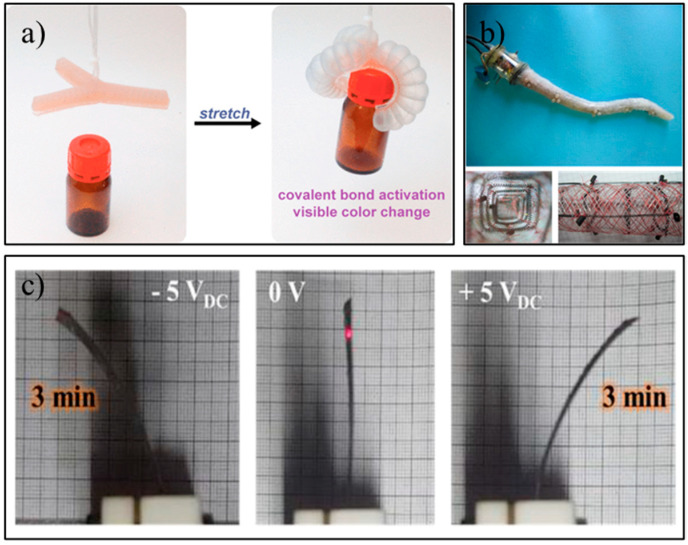
(**a**) An example of a PN soft-robotic gripper manipulating an object. The gripper also has color-changing ability due to covalent bond activation, courtesy of Gossweiler et al. [56] with permission obtained from ACS Publications; (**b**) an example of variable-length tendon-driven octopus-inspired robotic arm, which utilizes SMA springs in both the transverse and longitudinal direction; courtesy of Cianchetti et al. [61], open access article; and (**c**) examples of ionic EAP actuator motion under different DC voltages, courtesy of Park et al. [66], open access article.

Before realizing the potential of using pressurized actuation mechanisms in SARs, we must first explore their basic mechanisms. The pressurized fluidic and pneumatic-driven actuators operate based on the principle of pressure-induced structural expansion [42]. They are one of the most popular forms of soft actuation [46,53]. Generally, they consist of a single or multiple hollowed structures (often called Pneu-Nets or PN [54,55]) that are made from elastomeric polymer materials. By alternating the internal hollowed structures of the actuator with pneumatic or hydraulic pressure, motions, such as bending, twisting, expansion, or contraction, can be achieved. For example, Figure 2a [56] shows a PN soft-robotic gripper design bending its fingers when actuated and its capability to manipulate objects. The advantages of PN include that they (1) are light weight (depending on size, PN usually weigh between 1 and 100 g, not including the pressure pump) and (2) have less time delay than other material-based actuators, such as thermally triggered shape memory alloys or polymers and the omnipresence of air [42]. One of the limitations of pneumatic soft actuators is the requirement for hoses or tubes to be directly connected to the robots to supply the pressurized medium into the PN. To construct an untethered standalone soft robot that is not connected to an external pump, the controller electronics, mini air compressors, and the batteries will need to be integrated into the robotic design [51]. Furthermore, any puncture to the robot’s pressured chamber or leakage may be catastrophic for its movability [57]. To avoid such undesirable pressure changes, the wall thickness of the soft actuator needs to be appropriately designed so the chamber will be flexible enough to withstand the compressed air while thick enough to avoid bursting. A potential solution to possible punctures is to utilize self-healing materials to fabricate the PN [58]. This topic is discussed in the Self-Healing Properties Section below. 

#### 5.1.2. Variable-Length Tendon-Driven Actuation

As mentioned earlier, SAR motions can be achieved by using servo or DC motors at the joint. However, certain locations within the SAR, such as its head, may have limited space for fitting multiple motors for achieving individual control of all the facial features. To overcome such a challenge, variable-length tendon-driven actuation can be utilized as it allows for remote actuation over long distances within the SAR design. Such actuation mechanisms have already been implemented in soft robots as a key feature to achieve motion. In the design of soft robots, the tendons can be constructed from tension cables or shape memory alloy (SMA) actuators [42]. Such robots usually consist of multiple soft segments with the cable embedded inside. The cables are used to manipulate the segments with a controlled force to achieve a desired motion [59]. Furthermore, a granular medium, such as ground coffee, can be combined with the tension cable to achieve variable stiffness [60]. The granular medium has the ability to transition between a loose flowing grains phase and a rigid solid phase, depending on the particle’s packaging condition, whether individual particles are loosely touching each other or being tightly squeezed together in a condensed state [60]. When tension is applied to the cable, the granular medium is compressed within the soft segment and, therefore, changes the stiffness of the segment. As a result, bending and twisting motions can be achieved. Compared to the tension cables, SMA is a smart material that has the ability to “remember” an original shape and recover back to its pre-deformation when subjected to an external stimulus, such as heat [61]. The advantage of using variable-length tendon-driven actuation systems is that the cables can be operated over a long distance for the actuation of remote components. With the ability to fit between small gaps, the cables can be easily placed within an SAR if the robot has limited internal space. A major limitation of SMA is that the contraction strain produced by the SMA is relatively small (<5% of the initial length [61]). To overcome this shortcoming, SMAs can be made into spiral or coil shapes, which can enhance their deformation to over 300% [42]. Figure 2b [61] is an example of variable-length tendon-driven octopus-inspired robotic arm constructed from SMA springs. The SMA springs produce contraction motion once they are subjected to eclectically induced Joule heating. The contraction enables the soft robotic arm to achieve different motions, such as bending and twisting, that can be used for object manipulation.

#### 5.1.3. Electroactive Polymeric Actuators

Despite their unique movement behavior, electroactive polymers (EAPs) have yet to be implemented into SAR designs due to their actuation requirements, as previously mentioned, such as high voltage and the presence of aqueous environment [42]. EAPs are polymers that respond to an applied electric field by changing their geometries [62]. EAPs can be further categorized into two major groups [62,63]: (1) electronic EAP (driven by electric fields or Coulomb forces) and (2) ionic EAP (driven by the mobility or the diffusion of ions). Electronic EAP includes four different types of polymers [62]: ferroelectric polymers; liquid-crystal polymers (LCP); electrostrictive graft elastomers; and dielectric elastomer actuators (DEAs). In contrast, ionic EAPs consist of [62]: ionic polymer–metal composites (IPMCs); conductive polymers; ionic polymer gels; and carbon nanotubes. Due to the diverse research on EAPs, we only provide a brief discussion on DEAs and IPMCs, as they show the greatest promise to be used for SoftSARs. DEAs utilize electrostatic forces to produce movement. The advantages of DEAs include high force (up to 87N [64]), large strain (over 1000% of its initial length [65]), and fast actuation speed (in a range of milliseconds), but they require a high DC electrical field (>150V/μm) [42]. In comparison, IPMCs require less voltage (<5V [63]), which makes them more efficient and safer as an actuator. The typical construction of IPMC is made from sandwiching an electrolyte layer with two flexible electrodes, one as cathode and the other as anode. When a voltage is applied to an IPMC membrane, the hydrophilic cations within the IPMC move toward the cathode, which results in bending of the membrane [62]. Due to such a property, the movement direction can be achieved by adjusting the direction of the voltage, as shown in Figure 2c [66]. One of the limitations of IPMC is the water content and the condition of the electrolyte layer, as it is critical for controlling the ionic movement. If the electrolyte becomes dry over time, the IPMC can no longer be actuated. Due to the limitations in the material performance, it is unlikely that EAPs can be easily integrated with the SAR designs based on their current requirements. For the development of DEA, a major focus of research has been on lowering the voltage requirements for actuation and progress has been made from the initial kV range requirement down to 450 V [67]. Unfortunately, it is still too high for SARs as most of these robots operate between 5 V and 12 V [68]. For the IPMC, the evaporation of the internal electrolytes significantly reduces the lifetime of the material. As a result, novel coating materials, such as parylene and silicone rubber, can be utilized for minimizing evaporation [69]. These new research directions may provide new solutions for improving the performance of EAPs for future implementation within SAR.

**Table 2:** Summary of different soft-robotic technologies and their materials.

Study	Actuation Mechanism	Soft-Bodied Materials
[51]	Pneumatic	Silicone elastomer, Ecoflex (elastomer)
[54]	Pneumatic	Ecoflex (elastomer), Polydimethylsiloxane (Sylgard 184, PDMS)
[54]	Pneumatic	Elastosil M4601 (elastomer), Ecoflex (elastomer), Polydimethylsiloxane (Sylgard 184, PDMS)
[56]	Pneumatic	Ecoflex (elastomer), Polydimethylsiloxane (Sylgard 184, PDMS)
[57]	Pneumatic, with self-healing property	1,1’-(methylenedi-1,4-phenylene) bismaleimide (DPBM) & furfuryl glycidyl ether (FGE)
[59]	Variable-Length Tendons	Ultra-high-molecular-weight polyethylene (UHMWPE), SMA helical-shaped wires (SmartFlex)
[61]	Variable-Length Tendons	Polyethylene (PE), SMA helical-shaped wires (SmartFlex)
[64]	EAP-DEA	Silicone sheet (Wacker Elastosil, 50 μm thickness)
[65]	EAP-DEA	Acrylic elastomer (3M VHB4910)
[66]	EAP-IPMC	Nafion (N117, Dupont), Graphene (Nippon Ining), Gold nanowire (Nanopyxis Co.), Poly(3,4-ethylenedioxythiophene), polystyrene sulfonate (PEDOT:PSS, Heraeus)
[67]	EAP-DEA	Single-walled carbon nanotube (SWCNT, Sigma-Aldrich), PDMS (LSR 4305, Bluestar), Polyethylene terephthalate (PET, Dupont)
[69]	EAP-IPMC	Nafion (N117, Dupont), Platinum coated electrode

### 5.2. Soft Sensing

The main sensory systems that have been used in SAR design are 2D and 3D vision [70], microphones [71], and tactile sensors [71,72]. They enable SARs to engage in effective HRI. Vision is used to recognize and classify user and environment states, including the nonverbal expressions of users via facial expressions and gestures [73], whereas microphones with natural language processing software are used for user speech and utterance recognition [72]. Tactile sensors on a SAR’s body enable it to perceive physical touch as well as the amount of force during physical contact [8]. Soft sensors provide increased deformability and address the limitations of traditional rigid sensors by being easily incorporated within the flexible or soft-shell bodies of robots [72]. 

The development of soft sensors has mainly focused on robot awareness with respect to its location and behaviors, such as measuring robot position, the amount of deformation, and/or force [46]. Furthermore, the majority of soft sensors provide sensory information based on physical contact of their bodies, which mimic skin by being able to sense different stimuli, such as pressure and changes in temperature, referred to as electronic skin (e-skin) [74,75]. As the sensing mechanisms of soft sensors are based on the changes in the intrinsic material properties, multifunctional soft sensors with strain, pressure, bending, and/or vibration measurements can be developed [76,77]. In SAR development, research has mainly focused on the implementation of tactile sensors for a robot to interpret human intensions through physical touch. As a result, these sensors are generally used to measure only contact pressure, such as in Paro [35] and Kaspar [78]. With the utilization of a soft sensor, it is possible to obtain multiple sensory signals within a single-sensor component. Furthermore, soft sensors also offer advantages, such as flexibility (Young’s modulus close to human skin, 25–220 kPa [79]), stretchability (>100% strain [80]), and low thickness (ranging from a few micrometers to less than 1 mm [77]), which are more suitable for SARs, as traditional semi-conductor-based sensors lack flexibility [77]. Pressure, strain, and temperature modes are the three most applicable sensory modes in soft sensors that can be implemented in SoftSARs for HRI. As the underlining physical principles of flexible pressure and strain sensors are the same, they will be discussed together.

#### 5.2.1. Soft Pressure and Strain Sensors

The major difference between a soft pressure or strain sensor and their traditional rigid counterparts (such as a sensor made from lead zirconate titanate (PZT), a type of ceramic material that exhibits the piezoelectric effect [81]) is the use of polymer-based or low-stiffness materials, such as polydimethylsiloxane (PDMS) [82], to achieve overall flexibility and softness, which are important characterizations for applications, such as wearables and e-skins [74]. In these applications, the sensors may be subjected to deformations, such as stretching and bending; therefore, the material of the sensor is required to be flexible in nature. Similarly, the design of SoftSARs could consist of a “soft” exterior with such embedded flexible sensors for physical interactions.

Traditional capacitive pressure sensors measure pressure based on the change in the capacitance of a diaphragm, while the material-based soft pressure sensors are designed by replacing the diaphragm with a flexible dielectric layer placed in between two flexible conductive electrodes [83]. The major advantage of this particular soft sensor is the reduction in the sensor’s overall thickness, namely from a few millimeters (such as the rigid sensor described in [84]) down to hundreds of microns [74], along with improved softness and stretchability. Soft capacitive pressure sensors can be incorporated in SAR designs to measure user-applied pressure or force (correlated to change in capacitance) during physical interactions. Furthermore, soft capacitive sensors can also be used to monitor the robot’s motion, as changes in elongation also result in changes in the capacitance of the sensor [74]. Xu et al. [85] demonstrated the ability of a highly flexible and stretchable capacitive sensor that can be attached to different parts of human bodies, such as the posterior deltoid, wrist, and finger joints, for motion monitoring. For example, the sensor is able to detect the lowering and extending of a person’s arm. In SAR design, this sensor could potentially be implemented on the joints of the robot’s arm for movement tracking during HRI, including during different gesture and body-language displays.

Flexible piezoresistive sensors measure pressure or deformation based on the change in the resistance of a flexible substrate [86]. To fabricate such sensors, a common practice is to disperse conductive fillers into a flexible polymer matrix to form a conductive composite substrate with an internal conductive network [87]. When external deformation is applied to the composite substrate, disconnection/reconnection occurs at the fillers’ interfacial junctions, which results in changes in the resistance [87]. An advantage of such sensory design is the simplicity of manufacturing, as the sensor can be constructed with a single manufacturing step by dispersing the conductive filler with the polymer. The change in resistance can be correlated to the applied pressure or the elongation of the sensor. In SAR applications, a user’s physical interaction and the robot’s behavior can both be quantitatively measured by the soft piezoresistive sensor. Similar to the soft capacitive sensor, pressure resulting from pHRI, including hugging or handshaking, can be measured. The only difference between the soft capacitive and piezoresistive sensors is the output signal; namely, changes in the capacitance versus changes in resistance. 

The expected force/pressure during pHRI with SARs can be a result of people touching, hugging, petting, and/or holding the robots or the robots actively initiating these behaviors. Tsetserukou et al. [88] reported that human–human hugging can generate a pressure between 1.4 kPa and 5.9 kPa while Cepriá-Bernal et al. [89] reported that human hand grasping can create pressure between 30 kPa and 270 kPa. As a result, soft pressure sensors would need to detect a minimum pressure range from 1.4 kPa to a maximum of 270 kPa. Flexible capacitive pressure sensors are able to detect a wide range of pressures due to their high sensitivity, ranging from subtle pressure (1 Pa−1 kPa) to low (1 kPa−10 kPa) and medium (10 kPa−100 kPa) and even to high pressure (>100 kPa) [90]. During HRI, a SoftSAR could experience a low-pressure input from a user when engaged in handshaking or petting and a medium- to high-pressure input during hugging or hitting. In both cases, soft capacitive pressure sensors can be utilized.

#### 5.2.2. Soft Temperature Sensor

Sensing human body temperature may be useful for SARs engaged in pHRI, as research has shown that temperature is correlated to human emotions [91]. Emotions, such as anger, fear, happiness, and sadness, can be associated with the changes in temperature in the body and the limbs [91]. For example, a person experiencing happiness has a higher overall core body temperature when compared to negative emotions, while sadness is characterized as a decrease in temperature in the person’s hands and legs. Therefore, the ability to detect user body temperature during physical interactions, such as hugging, touching, or handshaking, may be valuable for SARs. In such scenarios, thermosensitive [92] or thermoelectric [93] sensors can be utilized. In thermoresistive sensing, the resistivity of the thermosensitive/thermoelectric material changes with temperature due to the change in the charge carrier density or the carrier’s mobility [74]. In thermoelectric sensing, a small current and voltage are generated once a change in temperature is detected [93]. An advantage of thermoelectric sensing is that a passive physical phenomenon is utilized; therefore, the sensors are self-powered and do not require an external power source [93], which further simplifies their design for SAR implementation.

### 5.3. Software and Hardware Specification Needs of SoftSARs

The computational requirements of the soft actuators and sensors need to be identified when developing SoftSARs. As the soft-robotics technology evolves with additional functionalities, such as complex movement control [94], advanced signal processing [67], and remote/untether control abilities [51], there is a need for more sophisticated control strategies and higher computational requirements. PNs are popular actuators for soft robots and can generally be controlled using miniature air-pressure valves. Pressure sensors can be used to monitor pressure and provide feedback to off-the-shelf microcontrollers and pneumatic hardware, where, for example, closed-loop PID control is used to control the output signals to the pressure valves. More-advanced soft actuators, including dielectric elastomer actuators (DEAs), presented in [67] also contain several off-the-shelf onboard electronics, such as transformers (ATB322515), MOSFETs (EPC2035, BSS127), diodes (CMSD2004S), photosensors (VCNT2020), and use a microcontroller (LPC1102) to implement a flyback circuit to control the charge/discharge cycle of the DEA actuator. These onboard electronics provide the necessary computational resources, which enable a soft robot to autonomously navigate on a surface with pre-printed paths. In another example, the untethered PN quadrupedal soft robot [51] also contains off-the-shelf mini air compressors (BTC IIS, Parker Systems), a microcontroller (ATmega168, Atmel Corporation), and an off-the-shelf camera (GoPro Hero2, Woodman Labs) which enable remote communication to a person with a live video and audio feed. For movement control, control programs can be written and stored in the onboard memory of the microcontroller using, for example, the Arduino interface for inputting direct commands for controlling the valves and air compressors such as inflating/deflating the soft robotic body to achieve required motions and actuation. 

With respect to soft sensing, recent research has considered the incorporation of various electronic components into the soft sensory design for creating wire-free [95] and battery-free [96] flexible devices. Due to the flexible nature of these soft sensors, they can be integrated with soft-robotic techniques for tactile and position sensing, as discussed in Section 5.2. For example, in [95], a flexible soft sensor used for monitoring gestures transmitted information of strain and deformation from hand gestures or finger movements using Bluetooth low-energy (BLE) system-on-a-chip (SoC, Nordic nRF51822). In addition to the advances in electronics, control systems have also been developed in recent soft-robotics research [97]. In [97], a closed-loop PID controller was designed for a soft glove system to provide real-time feedback of applied forces (by a human) and, thus, the soft actuators of the glove were able to generate a peak force in the safe region. 

In general, control strategies for soft robots differ from rigid SARs. Namely, the movements of rigid SARs are mainly defined by the rotation and bending of each joint connected by rigid bodies. However, soft robots are able to deform at all points across their soft materials, resulting in infinite DOFs [44]. Furthermore, since soft materials posses nonlinear and time-dependent material properties, model-based control approaches are difficult to use [98]. Model-free control methods include using machine learning (ML) or deep learning (DL) methods for both the actuation and sensing control of soft robotics [99,100]. For example, in [101], the local Gaussian Process Regression (GPR) learning method was used to estimate the motion of a PN soft actuator based on the robot’s kinematic model and its configurations obtained from sequential images of a camera mounted on the robot’s end-effector. In [102], ML methods, including k-Nearest Neighbors (kNNs), Support Vector Machines (SVMs), decision trees, Gaussian processes, and linear models, were used to calibrate and determine the bending/twisting behavior of a soft-robotic sensor embedded with multiple optical fibers. Such a soft sensor can be an important feature for SoftSAR in the future for determining robot hand and arm orientations. As SoftSARs will engage in pHRI with different users and potentially various common objects, soft sensors in combination wiht machine and deep learning methods can also be used for object classification. In [103], convolutional neural networks (CNNs) and Long Short-Term Memory (LSTM) networks have been used to classify objects grasped by a tactile glove containing a soft piezoresistive sensor array. With deep CNNs analyzing the pressure distribution within the sensor array, individual objects, such as a spoon, mug, and can, can be identified. 

Machine and deep learning approaches can also be used in controlling the actuation of soft robots. Mainly, input information, such as current states/positions of the soft actuator from internal sensor data or cameras (onboard or external), can be used as inputs into ML or DL models (feedforward neural networks (FNN), CNNs, etc.) to be mapped to control parameters (such as pressure input for a PN actuator and voltage applied for EAP) [99]. For example, in [101] the potential of motion estimation of a soft PN actuator with a local Gaussian Process Regression (GPR) learning method was presented. Ref. [104] presented a machine learning method with Bayesian optimization for the control of 3D-printed soft IPMC actuators in a soft crawling robot. Ref. [105] utilized deep reinforcement learning with a model-free method to achieve the dynamic feedback control of DEAs.

In general, SARs are becoming more intelligent in terms of perception and adaption to users and the assistive tasks they need to perform. Recently, the incorporation of ML and DL methods has been used for object or person classification in SARs [106], affect classification [107], and robot assistive behavior decision-making [108]. To-date, SARs have mainly used CPUs to meet the computational needs of their respective tasks when a SAR does not need to execute high computational algorithms and for cost reduction [70]. However, when incorporating algorithms, such as real-time object detection YOLO (You Only Look Once) and FCNN (Fully Convolutional Neural Network), to be executed together, it is necessary to use computer resources with greater-than-regular hardware (RAM and slow processors) and video graphic cards with GPUs [70]. Table 3 presents a number of existing SAR platforms with their corresponding onboard CPU and input/output computation requirements. Currently, only a few have incorporated embedded GPUs, such as NVIDIA Jetson, to allow for online training of their ML/DL methods [109].

## 6. A Roadmap for SoftSAR

As discussed in Section 5, of the emerging soft-robotic actuation (pneumatic, variable length tendons, and EAP) and sensing (soft pressure/strain/temperature sensors) devices, we believe soft-robotic technologies can be integrated into current SAR development. SAR research focuses on the development and deployment of assistive robots to help people using primarily social interactions via social cues and norms, with some robots promoting physical interactions as well. To obtain acceptance and an overall positive experience from users, SARs need to have the ability to engage in bidirectional communication. To be able to support the cognitive, social, and physical abilities of users, including those that are vulnerable, the display of SARs’ intentions and behaviors in assistive HRI scenarios is very important to the long-term use of these robots. This directly relates to the larger research challenge of endowing SARs with emotional intelligence. SARs, generally, also display emotions with their behaviors [113]. Nonverbal expression of emotions is widely accepted to convey affective and emotional information, as well as other important social interaction functions, including regulating turn-taking in conversations [114]. Using soft materials and actuators with new technologies, such as PN actuators with color-changing ability, as discussed in Section 5, SoftSARs have the potential to display emotions through the deformation of their “soft” outer shells or skin.

**Figure 3:**
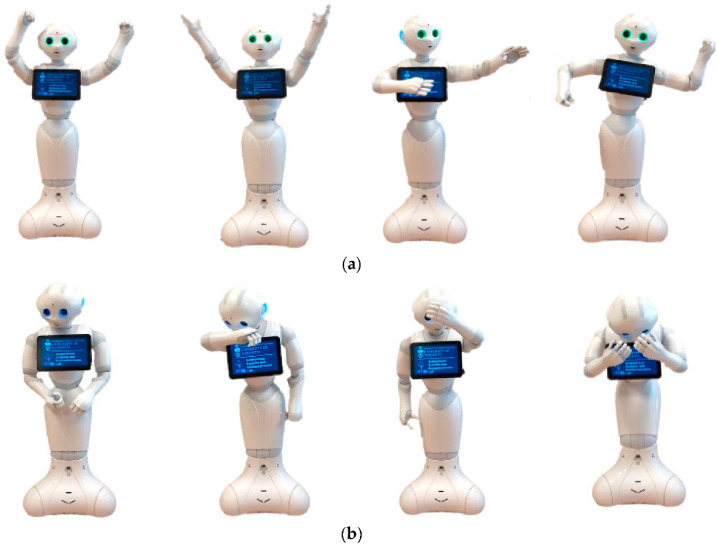
The Pepper robot displaying different emotions using nonverbal communication via its head, torso, and arm movements and changing eye colors: (**a**) positive valence and high arousal; and (**b**) negative valence and low arousal; courtesy of Shao et al. [116], open access article.

SARs can also communicate through physical interaction using movements of their entire body or body parts [28,115]. An example is shown in Figure 3, where the Pepper robot has the ability to communicate its emotions with users via moving its head, body, and arms or changing its eyes’ colors [116]. However, the safety of both the human and the robot is a big concern during pHRI, especially with vulnerable populations. To ensure safe physical interactions, common solutions utilized by current SAR designs include using soft and passive materials for the robot’s outer shells [26] or the implementation of complaint actuators and mechanisms [21], as described above. Compared to these design approaches, soft robotics can bring distinct solutions to reduce the safety risk during pHRI through the utilization of soft structures in combination with soft actuation and sensing throughout the body of the robot. Advances in the materials of soft robotics also promote new and unique characteristics, such as self-healing properties, that SoftSARs can take advantage of. Self-healing properties would be of interest to address the possible damage to a robot during repeated prolonged pHRI or harsh interactions from users.

There are unique engagement opportunities for both the soft-robotics and SARs research fields. Such technology integration has great potential to improve the HRI experience of users. For the soft-robotics community, researchers will have the opportunity to consider human-centred design in the design of soft structures and mechanisms, while gaining insight into HRI, assistive technologies, and human factors. The current soft-robotic research trends have mainly focused on using new materials or techniques for generating robotic motion and object manipulation. This new shift is important as soft robotics become deployed in real-world applications to help people and society through SoftSARs. 

We provide a roadmap for the future research directions for how soft-robotic actuation and sensing can be utilized to improve SAR design with respect to: (1) robot emotion and intent expressions through nonverbal communication means, (2) safe pHRI, and (3) self-healing properties.

### 6.1. Emotion and Intent Expressions through Nonverbal Communication Means

Nonverbal communication in social HRI is crucial for engagement as it represents intuitive interactions [11,117] and can be used to transmit both emotional and functional intent [118,119]. Furthermore, it can be used to augment verbal communication by reinforcing what is being said [120]. Emotions influence decision making and behaviors of people and can be used to develop robot emotional intelligence [113,121,122]. SARs should be able to infer human emotions and also express their own emotions [120]. Nonverbal communication by SARs can be achieved using various modes that resemble human-like features, such as facial expressions [14,72], head and arm gestures [123], body postures and movements [124], eye gaze [125], to animal and creature-like behaviors with changes in color [126], vibrations [127], and skin texture [115].

SARs use movements of facial features to mimic facial expressions [14,28]. However, to achieve independent control of different facial components, such as the lips, eyelids, and eyebrows, a large number of motors is required. For example, the humanoid robotic face, Eva [128], has a total of 25 servomotors in order to display different facial expressions; the Albert Einstein HUBO [129] robot has 31 servomotors in its head and 35 servomotors in its body. Furthermore, the Erica robot [130] utilizes a total of 44 pneumatic actuators and Geminoid HI-2 [131] has a total of 50 pneumatic actuators. It should be noted that the pneumatic actuators utilized in [130,131] are not the soft actuators discussed in this article. These pneumatic actuators are commercially available metallic air cylinder actuators [130,131] and can only provide single-directional actuation (linear expansion or rotation), while the PNs are composed of flexible polymeric material that can undergo both surface and volumetric expansion, as discussed in Section 5.1.1. We believe the utilization of PN may be used to simplify robot face design. For example, the facial components that require actuation can be connected with a single network of multiple pneumatic actuators while controllable pressure valves serve as the junctions between individual actuators. To express different emotions, the valves will only allow certain actuators to be pressurized; therefore, facial expressions can be achieved with a single PN network rather than multiple individual actuators. 

The same design framework can also be applied to other body features, such as arms or hands, to provide robot body language during HRI. For example, a SoftSAR’s hand can be designed with a single PN containing five independent pressure valve controllers for the activation of individual fingers for gesturing. As a result, PNs can reduce the number of actuators required. 

In addition to facial expressions and body language, colors are known to be associated with different emotional states [132]. For example, red is usually associated with intense emotions, such as anger, while blue is associated with sadness [132]. Furthermore, positive emotions are related to light colors, such as yellow [132]. Robots display different emotional colors through the use of LEDs [133]. However, SARs can potentially use color-changing PN material to achieve color changes on the surface of their bodies [56], as shown in Figure 2a. In addition to the ability to deform their shape, as discussed in Section 5.1.1, the PNs reported in [56] have color-changing ability as the material is constructed from molecules (spiropyran mechanophore) with covalent bond activation that can respond to physical deformation. As the PN is inflated, the expansion applies microscopic molecular deformation within the material and results in the macroscopically observable coloration. In addition, colored fluid can also be utilized within the PN [134] to achieve this ability. As the PN is constructed from semi-transparent materials, the color of the PN can be changed by passing color fluid into the network directly [134]. This allows an emotion to be expressed with both color changes and soft actuation (e.g., facial or body expressions) at the same time. For instance, a color-changing PN containing pink fluid can be placed in the cheeks of the SoftSAR for simulating happiness or excitement while, simultaneously, the cheeks can inflate or the corners of the mouth can move upwards through PN expansion.

Physical interaction can also be an effective form of nonverbal communication. In fact, it has been shown that physical touch from a robot can actually promote positive emotions in humans [135]. During such interactions, soft pressure sensors, such as the soft piezoresistive sensors discussed in Section 5.2.1, can provide valuable information for SoftSAR. The biggest advantage of using soft sensors is their flexibility and adaptability. Due to limitations in their physical sizes, traditional rigid pressure sensors have only been installed in more spacious areas of SARs, such as the chest or arms [84]. The skin-like soft pressure sensors can significantly reduce the required sensing space and can be easily integrated in areas where space is limited, such as the hands and fingers of a robot. Flexible capacitive or piezoresistive sensors can be investigated for measuring the contact forces exerted by humans along with soft thermoelectric sensors in order to classify and infer human emotions through multi-modal contact soft sensing. 

The utilization of soft-robotic technologies also enables SoftSARs to express varying emotions during both sHRI and pHRI, providing a unique interaction experience. Pena et al. [136] designed and validated [137,138] a robot that utilizes body temperature and facial expressions to communicate its emotional expression and emotional state. The robot has the ability to change its surface skin temperature between 10 and 55 °C while displaying different facial expressions, such as joy, neutrality, anger, sadness, and fear. The results indicated that users focus on the robot’s facial expressions to interpret the robot’s emotional expressions; however, when judging the overall emotional state of the robot, the change in its body temperature played an important role. As a result, the group concluded that a combination of facial and thermal expressions is a necessary approach for the robot to communicate its emotions. Based on such a finding, SoftSARs can also achieve a warm surface body temperature by passing warm temperature fluid though PNs [134], which enables them to adjust their body temperature when expressing different emotional states while they display different facial expressions via PN actuation. 

Lastly, dielectric EAPs (discussed in Section 5.1.3) have the potential to be utilized to produce vibrations in a robot, due to their fast response time. Such behavior can be associated with a SoftSAR expressing intense emotions, such as surprise or agitation. However, since the currently available dielectric EAPs require a high voltage source (450 V [67]) for operation, which is considered too high for the operation of SARs as they require a voltage source between 5 V and 12 V [68], further research developments in dielectric EAPs are needed.

### 6.2. Safe Physical HRI

Many HRI scenarios promote close contact between a robot and a human [9], especially for cognitive and sensory therapies [21]. In these scenarios, the SARs are covered with soft or elastic material to absorb any possible impacts due to contact forces from users, in order to protect both the human and the robot. Passive compliant mechanisms can also be used to ensure the actuation motion generated by the SAR will not harm the users [21]. In terms of soft actuation, the PNs discussed in Section 5.1.1 can be adapted to further enhance the safety performance of the SAR. As PNs are constructed from elastic materials and filled with air, they have the ability to dissipate impact forces applied from users, therefore, ensuring minimum forces are transmitted back to the human. The variable-length tendon actuation system discussed in Section 5.1.2 can also be implemented in SoftSARs in combination with the passive compliance mechanism to enhance safety [29] due to the similarity in their design. Both variable-length tendons and passive compliance mechanisms [29] feature long cables, while the stiffness of the cables can be adjusted based on the tension in the cables. During pHRI associated with high forces (i.e., hitting), the adjustable stiffness allows these forces to be absorbed. 

The next-generation SoftSARs can also adapt dynamic inflate/deflate behaviors into their design, due to the inflatable property of PNs. Air pressure can be regulated with a control system that takes into account different impact situations to control a PN to act as a shock absorber. Furthermore, the inflatable properties suggest that a PN can withstand multiple and continuous impacts by adjusting its internal pressure, under the assumption that no punctures occur from the impact. From a sensory perspective, the utilization of soft sensing can also be used to monitor physical interactions and ensure safety, by combing flexible pressure sensors with PNs to provide sensing feedback to the controllers of the PNs. 

### 6.3. Self-Healing Properties

SARs may suffer damage, such as scratches and tears, from prolonged or repeated use. For example, users may exhibit aggressive behaviors towards the robots, due to anger, agitation, or increased stress levels [139,140]. A unique solution for SoftSARs is the utilization of self-healing materials in the design of their outer shells. There are two different types of self-healing materials: (1) self-healing agent filled-microcapsule design [141] or (2) the utilization of dynamic bond formation within the material, such as hydrogen bonding [142,143] or ionic interactions [144,145,146]. In microcapsule design, mechanical damage (i.e., scratches) on the robot’s surface will break the microcapsules and release a self-healing agent (for example, dicyclopentadiene (DCPD) [147]) to repair the damage. Currently, there are multiple material choices for fabricating the microcapsule with biocompatible options [147]; therefore, people can safely interact with SARs containing these materials. For the self-healing materials that utilize dynamic bond formation, scratches/tears break the polymer chains within the material physically. Once the damage occurs, the polymer chains will slowly diffuse into the damage location and reform the chemical bonds, therefore, healing the surface. Similar to microcapsule-based materials, dynamic bonding self-healing materials can be made from biocompatible polymers [148]. In both types of self-healing materials, the material can be considered as “autonomous self-healing” if no external stimuli or energy input is required. However, to-date, the majority of self-healing materials require an external stimulus, such as heat, light, or chemical reaction, in order to initiate the self-healing process [149]. 

As PN is the most promising soft-robotic technology to be utilized in SARs, a self-healing material that can be fabricated into PN would be of interest. Terryn et al. [57] created a self-healable soft PN actuator by adapting the Diels–Alder (DA) reaction between a diene (furan) and a dienophile (maleimide) monomer, which created thermoreversible DA cross-links in the polymer network. If the material is damaged, an external heat (>70 °C) stimulus can be used to trigger the self-healing process. At the damaged location, DA bonds are mechanically broken. The external heating disturbs the equilibrium and results in the formation of additional furan and maleimide functional groups. With an increase in temperature, the mobility of the polymer chain increases and functional groups are slowly migrating to the damaged site. By applying controlled cooling, the DA bonds are slowly reformed within the material; thus, a self-healing behavior at the macroscopic level can be observed and a damaged PN can be re-inflated.

## 7. Challenges for SoftSAR

There are new possibilities and opportunities that soft-robotic technologies can provide SARs, mainly due the introduction of soft materials to address SAR capabilities for HRI using soft smart structures, actuation, and sensing. Furthermore, SoftSAR design can lead to human-friendly and safe robot interactions. However, to achieve these, a number of open technical and social challenges need to be addressed in SoftSAR design, including: (1) barriers to technology implementation, (2) user perceptions and acceptance, and (3) large-scale deployment and commercialization.

### 7.1. Barriers to Technology Implementation

In order to construct the next generation in SoftSAR, it can be expected that novel materials need to be developed to meet the functional and safety requirements. As most of the current soft-robotic studies focus on small-scale demonstrations with robot sizes ranging from a few millimetres to centimetres [52], the same technologies will need to be scaled up to meet the requirements of larger-sized SARs, which are typically in a range of tens of centimetres (Paro [150] is 57 cm in length) to over a meter (Brian is 1.5 m in height). For such robots, the current elastic materials may not have the same actuation performance. A larger PN would require both a thicker PN exterior layer and a material with higher elastic modulus in response to a higher pneumatic pressure during operation. For example, different grades of silicone need to be considered to increase the elastic modulus [51]. Due to such changes, the response time and actuation force output will need to be tested to ensure that the desired performance can be achieved. Furthermore, many of the fabrication techniques, such as microfabrication, may no longer be applicable, due to the larger scale. 

### 7.2. Perceptions and Acceptance

Perceptions and acceptance of users will need to be investigated regarding the deployment of SoftSARs to explore their HRI benefits. There has only been one study that investigated the influence of soft-bodied SAR robots on user perceptions. In Saldien et al. [38], the Godspeed questionnaire was used to explore any cross-cultural differences in overall perceptions of the Probo robot between individuals from China, Romania, and Belgium. In particular, animacy and likeability of the robot had positive results, with no significant differences between the three groups. Other crucial appearance attributes, such as level of abstraction of expressions/movements and embodiment form (human-like, creature-like, or animal-like), should be considered when investigating preferences and acceptance of SoftSAR robots. One open challenge is to compare the effects of soft actuation and soft body separately for SoftSARs to see which one (if any) has a greater impact on user experience and acceptance of SoftSARs, or if it is the combination of the two that is important.

A few non-SAR studies have shown the importance of using soft skin for HRI. For example, in Jorgensen et al. [151], two silicone-based pneumatically actuated soft-tentacle robots (one more soft than the other) and a rigid tentacle robot were compared to determine if soft robots are perceived as more natural than rigid robots. Results found that users did not find the soft robots as more natural or appealing than the rigid robot, but the naturalness of touch and appeal was correlated with the softer robot. Furthermore, it was concluded that the natural perception of soft robots can vary from person to person [151]. In [152], the potential development of emotional expressions for a social robot was investigated. Cast elastomer and inextensible film layers were used to create spikes and goosebumps with skin texture. The study found that these texture expressions can be perceived by people as different emotions, regardless of visual or touch interaction modes, and, hence, can increase human perception of robot emotions. Therefore, it would be interesting to explore if using touch via soft skin in HRI with SoftSARs has important additional benefits to intent to use and uptake of such robots as assistants. Furthermore, investigating how touch in pHRI with such robots directly impacts affect and moods as well as wellbeing of users over rigid SARs is valuable.

### 7.3. Large-Scale Deployment and Commercialization

Soft-robotic technologies are still in their early R&D phases and the market-ready soft-robotic industries are still maturing. There is no doubt that the development of soft robotics is moving at an accelerated pace in academia; nevertheless, the corresponding commercial sector has yet to be established. In such circumstances, the development of SoftSARs should grow alongside the soft-robotics industries as an important application of these technologies. As an outcome of a cross-disciplinary research, we strongly believe the development of SoftSARs will soon be ready for use in healthcare settings (for cognitive and social therapy and to support wellness and aging in place) as new materials and manufacturing techniques arise from the field. 

### 7.4. The Potential Tactile Ethics of SoftSAR

As pHRI is an important aspect of SoftSAR, the softness or hardness of materials for building such SoftSARs needs to be carefully considered. During pHRI, tactile interaction would be unavoidable; thus, it is important to balance SoftSAR tactile engagement against emotional manipulation of users [153]. As discussed in Section 2, Paro the seal robot [140] uses its soft and plush surface for enhancing therapeutic effectiveness [154]. However, an important question to be considered would be whether such effectiveness should be measured by the caregivers or the users themselves [153]. With the development of SoftSAR, similar ethical questions and implications may also arise. 

It should also be noted that the proposed SoftSAR will likely be constructed with soft and flexible materials. However, whether such physical soft presence would affect how people view the robot needs to be considered. Due to its soft appearance, a robot can draw more aggressive, reckless, or addictive touch instead of the type of interactions the robot was initially designed for [153]. As explained in [155], cute and cuddly objects can actually excite an aggressive response. If the SoftSAR was also designed to feature a cute appearance and soft materials, will it be prone to being squeezed/hugged too much, thus, affecting the wellbeing and the mindset of the users?

With the advances in both HRI and SAR research, it has been discovered that people can actually attribute feelings and personal qualities to a robot, despite the fact that people are aware that the robot cannot reciprocate these feelings [156]. With a softer exterior and promotion of tactile interactions, SoftSARs may be pleasing or comforting to a person, which may further elicit a response of trust and openness. Thus, it is essential for researchers to carefully consider both the softness feature of SoftSARs and specific HRI scenarios that can contribute to building a bonding relationship between SoftSARs and users. 

## 8. Conclusions

We introduced the cross-disciplinary research field of SoftSAR and how the disciplines of soft robotics and socially assistive robots can benefit from cutting-edge collaboration. We see SARs as an important emerging robotic application for soft-robotic technologies and provided a research roadmap in how to bridge the gap between these two fields by highlighting new research and development possibilities and future novel research directions. We see a future society with many examples of functioning SoftSAR systems deployed to help people in everyday life.

## Figures and Tables

**Table 1:** Traditional robots vs soft robots.

	Traditional Robots	Soft Robots
Core design	Stiff structures and linkage May have compliant linkage Soft material may be used to cover internal structures	Made entirely from soft and low compliant materials No rigid linkage
Movement control	Motors or linear actuators	Smart material based responsive actuation mechanism (pneumatic, shape memory effect, electroactive polymers, etc.)
Overall shape and geometry	A pre-defined shape that cannot be deformed	Highly flexible and deformable
Degrees-of-freedom (DOFs)	Limited by the number of joints and linkages	Continuum design enables close to infinite DOFs

**Table 3:** Computational specifications of different existing SARs.

SAR	CPU	Input/Output Computational Needs
Paro [110]	32 bit RISC	Posture sensor, temperature sensor, light sensor, tactile sensors, microphones, loudspeakers, motors
Brian [14]	1.8 GHz Intel i5 3337U	Nintendo Wii Remote IR Sensors, 10-MP Creative Webcam, Kinect Sensor, loudspeakers, servo motors
Kasper [78]	LynxMotion SSC32 Servo controller board (cf. LynxMotion 2007) with USB connection to PC	Video cameras, loudspeakers, microphone, RC servos (for movement in head and arms)
Pepper [111]	1.91 GHz. Intel Atom E3845	Six-axis inertial measurement unit; microphones, 2D cameras, 3D sensors, tactile sensors, bumpers, laser sensing modules, loudspeakers, sonar sensors, infrared sensors, network connectivity modules, tablet, DC motors
Nao [112]	500 MHz AMD GEODE	Tactile sensors, video cameras, gyroscope, accelerometers, infrared sensors, bumpers, ultrasonic sensors, loudspeakers, microphones, DC motors

## Data Availability

Not applicable.
